# Deep-Tissue Photothermal Therapy Using Laser Illumination at NIR-IIa Window

**DOI:** 10.1007/s40820-020-0378-6

**Published:** 2020-01-24

**Authors:** Xunzhi Wu, Yongkuan Suo, Hui Shi, Ruiqi Liu, Fengxia Wu, Tingzhong Wang, Lina Ma, Hongguang Liu, Zhen Cheng

**Affiliations:** 1 Institute of Molecular Medicine, College of Life and Health Sciences, Northeastern University, Shenyang, 110000 People’s Republic of China; 2 Department of Neurosurgery, The Fourth Affiliated Hospital of China Medical University, Shenyang, 110000 People’s Republic of China; 3 Changchun Institute of Applied Chemistry, Chinese Academy of Science, Changchun, 130000 People’s Republic of China; 4 Molecular Imaging Program at Stanford, Stanford University, Palo Alto, CA 94301 USA

**Keywords:** Photothermal therapy, Deep-tissue, NIR-IIa, 1275 nm laser, Molecular imaging

## Abstract

Photothermal therapy (PTT) using near-infrared (NIR) light for tumor treatment has triggered extensive attentions because of its advantages of noninvasion and convenience. The current research on PTT usually uses lasers in the first NIR window (NIR-I; 700–900 nm) as irradiation source. However, the second NIR window (NIR-II; 1000–1700 nm) especially NIR-IIa window (1300–1400 nm) is considered much more promising in diagnosis and treatment as its superiority in penetration depth and maximum permissible exposure over NIR-I window. Hereby, we propose the use of laser excitation at 1275 nm, which is approved by Food and Drug Administration for physical therapy, as an attractive technique for PTT to balance of tissue absorption and scattering with water absorption. Specifically, CuS-PEG nanoparticles with similar absorption values at 1275 and 808 nm, a conventional NIR-I window for PTT, were synthesized as PTT agents and a comparison platform, to explore the potential of 1275 and 808 nm lasers for PTT, especially in deep-tissue settings. The results showed that 1275 nm laser was practicable in PTT. It exhibited much more desirable outcomes in cell ablation in vitro and deep-tissue antitumor capabilities in vivo compared to that of 808 nm laser. NIR-IIa laser illumination is superior to NIR-I laser for deep-tissue PTT, and shows high potential to improve the PTT outcome.

**Electronic supplementary material:**

The online version of this article (10.1007/s40820-020-0378-6) contains supplementary material, which is available to authorized users.

## Highlights

- The laser of 1275 nm exhibited excellent ablation of tumors with 5-mm porcine muscle tissue blocking, while 808 nm laser failed.
- Polyethylene glycol-stabilized copper sulfide nanoparticles with similar absorption efficiency at wavelength of 808 and 1275 nm were employed to compare the potential of these two lasers in deep-tissue photothermal therapy.

**Electronic supplementary material:**

The online version of this article (10.1007/s40820-020-0378-6) contains supplementary material, which is available to authorized users.

## Graphical abstract

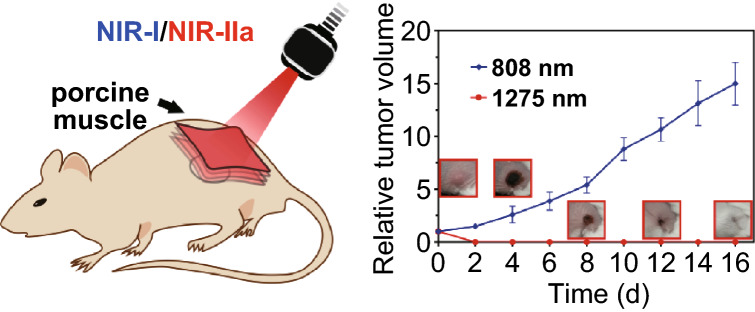

## Introduction

Photothermal therapy (PTT) is a novel therapeutic method for treatment of diseases especially tumor, which employs photo-absorbers to generate heat with irradiation of light. Compared to traditional therapeutic modalities, PTT displays excellent performance in tumor treatment owing to not only high specificity but also precise spatial and temporal selectivity [1]. The crucial factors that determine the PTT outcomes of solid tumors are mainly the absorption of photothermal transduction agents (PTAs) and the penetration depth of irradiation into tumor tissue [2–4]. Though great efforts have been devoted to increase the photothermal conversion efficiencies of PTAs, current PTT applications are still only suited for superficial tumors because of the limited penetration depth of light illumination [4–6]. To promote the application of PTT in the clinic, especially PTT for the treatment of deep-seated tumor, the penetration depth of the irradiation laser is expected to be improved.

The laser wavelength in the near-infrared (NIR) range is highly desired to be used in PTT because of less scattering and absorption by the tissues, which endow deeper penetration in comparison with visible light [7]. Two biological transparency windows are located in NIR region: the first NIR window (NIR-I; 700–900 nm) and the second NIR window (NIR-II; 1000–1700 nm) [1]. Recently, the application of NIR-II light as either emission or excitation [8–10] source for optical imaging or PTT, respectively, is receiving more and more attentions because of the intrinsic merits of light in the NIR-II window, including even deeper penetration depth and higher maximum permissible exposure (MPE) over NIR-I [11–14]. These advantages imply that NIR-II laser may be superior to NIR-I for deep-tissue treatment, which have been demonstrated by several studies recently. But all of these studies mainly focus on the comparison between 808 and 1064 nm laser [6, 15–17]. Laser excitation with wavelength longer than 1064 nm has not been explored for treatment.

NIR-II laser with wavelength between 1100 and 1400 nm possesses inviting prospect. In some recent research, using lasers of optical sub-windows such as 1300–1400 nm (termed as NIR-IIa window) and 1500–1700 nm (termed as NIR-IIb window) has been explored for better performance of fluorescence imaging [1, 18–21]. Spectra range of 1300–1400 nm is considered to be preferable to 1500–1700 nm since it avoids the increase in light absorption by water vibrational overtone over ~ 1.4 μm [18]. The choice of light at NIR-IIa also minimizes the adverse photon scattering effects in brain tissue. More importantly, laser with 1275 nm wavelength has been approved by the Food and Drug Administration (FDA) to be applied for physical therapy. It produces long-lasting beneficial effects for the treatment of chronic pain and fibromyalgia [22, 23]. As a laser with wavelength very close to NIR-IIa window, it may serve as a promising candidate for deep-tissue PTT. Therefore, in this study, the capacity of 1275 nm laser for PTT was investigated. Laser with 808 nm wavelength, which is most widely studied in PTT, was selected as a control wavelength for comparison. Polyethylene glycol (PEG)-stabilized copper sulfide nanoparticles (CuS NPs), which possess similar absorption efficiency at 808 and 1275 nm, were employed as a PTT agent and excited by either 1275 or 808 nm laser for in vitro and in vivo studies. For the first time, 1275 nm laser was evaluated for PTT in a xenograft tumor mouse model.

## Experimental Section

### Synthesis and Characterization of CuS NPs

CuS NPs were synthesized based on the method published before with slight modification [24]. In brief, 24 mg of Na_2_S·9H_2_O was added into 25 mL of an aqueous solution dissolved CuCl_2_ (13 mg) and sodium citrate (20 mg) under stirring at room temperature. Five minutes later, the reaction mixture was heated to 90 °C and stirred for another 15 min. Then, the mixture was transferred to ice-cold water. For purification, unreacted ingredients were dialyzed out through dialysis membranes (10 kDa).

TEM image was performed by using a JEM-2100 transmission electron microscope (JEOL Ltd, Japan) with an acceleration voltage of 200 kV. The hydrodynamic particle size distribution and zeta potential of CuS NPs were determined by using a Malvern Mastersizer 2000 (Malvern Instruments Ltd, UK).

### Synthesis and Characterization of CuS-PEG NPs

The PEGylation of CuS NPs was based on the method published before with some modification [25]. Briefly, 2 mg of methoxy-PEG-thiol (PEG-SH; 5 kDa) was added into 5 mL of CuS NPs solution. The reaction was allowed to proceed for 36 h at 4 °C. The reaction liquid was filtered through a 0.22 μm filter membrane first and was then centrifuged at 6000 rpm for 2 min using ultra-centrifugal filter units (50 kDa) to remove unreacted PEG-SH.

To evaluate the stability of CuS-PEG NPs in fetal bovine serum (FBS), CuS-PEG NPs were preserved in 50% FBS at 37 °C and the sizes of CuS-PEG NPs were determined by DLS in the following 7 days. To determine the relationship between PA signals and concentrations of CuS-PEG NPs, 1 mL of CuS-PEG NPs solution with different concentrations (0.125, 0.25, 0.5, 1, and 2 mg mL^−1^) were added into 4 mL centrifuge tubes. Then, the PA signals were detected under a 950 nm laser. Absorption spectra were recorded by using a Cary 5000 spectrophotometer (Agilent Technologies, CA, USA) with a 1.0 cm quartz cell.

### Comparison of Tissue Penetration Capabilities

Laser with wavelength of 808 or 1275 nm (QPC Lasers Inc. Sylmar, California) was conducted to pass through porcine muscle tissues of a series of thicknesses (2, 4, 6, 8, and 10 mm). Laser intensities before and after tissue blocking were recorded by a near-infrared spectrometer (NIRQuest512, Ocean Optics, Inc).

### Comparison of Tissue Scattering Effects to 808 and 1275 nm Laser

Porcine muscle tissues (2.5 cm long, 2.5 cm wide, and 0.8 cm in thickness) were laid flat. Different dosages of 808 or 1275 nm laser were conducted to irradiate at the center of the sagittal side of meat for 2 min to make similar temperature rise of meat. Thermal images of sagittal and axial sides of meat were taken before and after irradiation by an infrared thermal imager (FLIR T430sc, USA).

### Test of Heating Effect to CuS-PEG NPs Without Tissue Blocking

For “808 nm” group, CuS-PEG NPs solution with concentration of 0.25 mg mL^−1^ was plotted in 96-well plates, while for “1275 nm” group, the concentration of CuS-PEG NPs was 0.30 mg mL^−1^. The concentration of CuS-PEG NPs solution for each group is determined by a concentration correction experiment (Fig. S1). Then, 808 or 1275 nm laser with power density of 0.33 or 1 W cm^−2^ was conducted for 5 min. The temperature rises of CuS-PEG NPs in each well were calculated by the temperature rise values of the solution minus those of the solvent, deionized water.

### Comparison of Photothermal Heating Capabilities In Vitro

To compare photothermal heating capabilities between 808 and 1275 nm laser in deep-tissue environment, CuS-PEG NPs solution was added into 96-well plates with different concentrations for 808 and 1275 nm laser groups as mentioned above. Then, the wells were covered by porcine muscle tissues of different thicknesses (2, 5, 10, 15, and 20 mm), followed by exposure to 808 or 1275 nm laser with different power densities of 0.33 or 1 W cm^−2^ for 5 min. Temperature changes of CuS-PEG NPs solution of different groups were recorded by a thermal imager.

### Cell Line and Cell Culture

4T1 mouse breast cancer cell line was obtained from the Chinese Academy of Medical Sciences (Beijing, China), and DMEM cell culture medium was purchased from GIBCO (BRL, Rockville, MD, USA). 4T1 cells were cultured in DMEM (10% FBS and 1% penicillin/streptomycin) with 5% CO_2_ at 37 °C in a humidified incubator. Cells were generally harvested by treatment with 0.25% trypsin–EDTA solution.

### Comparison of Photothermal Capabilities In Vitro

In brief, 4T1 cells were seeded in a 96-well plate with 2 × 10^4^ cells per well and incubated at 37 °C in an atmosphere of 5% CO_2_ and 95% air overnight. Then, the culture medium was removed, and cells were rinsed with PBS. Then, 60 μL of PBS with or without CuS-PEG NPs (0.5 mg mL^−1^) was added into the wells. The wells were covered by porcine muscle tissues of different thicknesses (0, 2, 5, and 10 mm), followed by exposure to 808 or 1275 nm laser of 1 W cm^−2^ for 5 min. The wells were then rinsed with PBS and 200 μL of fresh medium with 10% FBS was added to each well. After incubation for another 6 h, cell viability was evaluated by a standard MTT assay. For cells staining, cells after different PTT treatments were rinsed with PBS, and 1 μL of propidium iodide (PI) and 15 μL of Hoechst 33,342 were added and incubated for 10 min. Then, cells were observed by fluorescence microscope with the excitation of 352 nm laser for Hoechst 33,342 and 488 nm laser for PI.

### Tumor Model

To establish tumor model, 3 × 10^6^ 4T1 cells suspended in 100 μL of serum-free DMEM were injected subcutaneously into the left back of mice. The tumor sizes were measured by a Vernier caliper and calculated as the volume = (tumor length) × (tumor width)^2^/2. Animal care followed institutional guidelines, and all experiments were approved by the local animal research authorities.

### Comparison of Deep-Tissue PTT Capabilities In Vivo

PTT was carried out when the tumor volume reached ≈ 60 mm^3^. Before laser irradiation, mice were anesthetized with 2% isoflurane using a MATRX VIP 3000 anesthesia machine. Then, CuS-PEG NPs (50 μL, 1 mg mL^−1^) or PBS (1 × , pH = 7.4) were intratumorally injected into tumors. The tumors were covered by 5-mm-thick porcine muscle tissues and then exposed to 808 or 1275 nm laser with power density of 1 W cm^−2^ for 5 min. After treatment, tumor sizes and mice weights were recorded every other day. The photographs of mice were also taken. On day 16, mice were sacrificed and tumors weights were recorded. There were three mice for each treatment group.

### Accumulation Study of CuS-PEG NPs in Tumor Through PAI

PAI was performed at different time points to monitor the accumulation of CuS-PEG NPs in tumors before and after CuS-PEG NPs (200 μL, 2 mg mL^−1^) were intravenously injected into mice (i.e., pre, 1, 2, 3, 6, 12, and 24 h). PA signals were detected under a laser of 950 nm.

### PTT with 1275 nm Laser

When tumors volume reached ≈ 60 mm^3^, mice (*n* = 3 per group) were anesthetized and injected with CuS-PEG NPs (200 μL, 2 mg mL^−1^) or PBS through tail vein. PTT with 1275 nm laser (0.2 W cm^−2^, 5 min) was performed 2-h post-injection. The temperatures of tumors were recorded during irradiation by an infrared camera. Tumor sizes, mice weights, and photographs were also collected after treatment as the same that mentioned in Sect. 2.10.

### Histology Analysis

To observe the histological change of tumor after PTT, the tumor tissues were excised from mice after PTT treatment and fixed with paraformaldehyde, dehydrated, sliced into 5-mm sections and subjected to H&E staining assay.

### Statistical Method

Measurement data were expressed as the mean ± standard deviation (SD). Statistical analyses of the data were performed with GraphPad Prism 7.0 (GraphRad Software, Inc., San Diego, CA, USA). A Student’s *t* test was applied to identify significant differences between groups.

## Results and Discussion

### Synthesis and Characterization of CuS NPs and CuS-PEG NPs

**Fig. 1:**
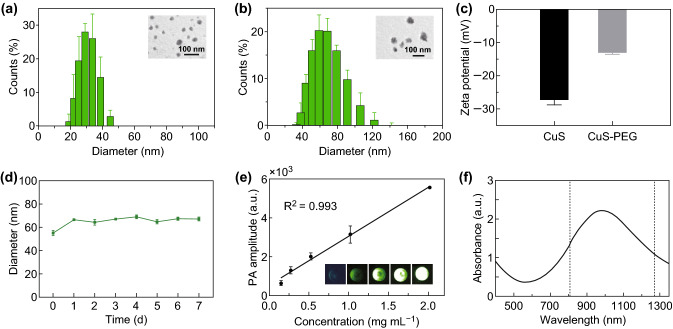
Characterization of CuS NPs and CuS-PEG NPs. **a** Morphology and size of CuS NPs. **b** Morphology and size of CuS-PEG NPs. **c** Apparent zeta potential of CuS NPs and CuS-PEG NPs. **d** Diameters of CuS-PEG NPs stored in FBS. **e** Linear fitting plots of PA amplitudes versus the concentration of CuS-PEG NPs under NIR laser irradiation. The inset is the PA image of CuS-PEG solution with different concentrations. **f** Absorption spectrum of CuS-PEG NPs. The error bars represent standard deviations (*n* = 3 per group)

In the study of Li et al. [24], they successfully synthesized [^64^Cu]CuS nanoparticles with small diameter (~ 11 nm) and strong NIR absorption which is suitable for molecular imaging and PTT. The absorption values of this type of nanoparticles at wavelengths of 808 and 1275 nm are relatively close, suggesting that it is suitable for comparison of PTT efficacy using NIR-I or NIR-II laser excitation. Moreover, as previously reported, for nanoparticles with diameter between 5 and 100 nm, lager nanoparticles generally show better tumor accumulation and longer retention than smaller nanoparticles, because of their favored enhanced permeation and retention (EPR) effect [26]. Therefore, in this study, larger-sized CuS NPs were synthesized according to the method published before [27] with a modification. TEM was used to evaluate the morphology of CuS NPs. As illustrated in Fig. 1a, CuS NPs were found to be well-dispersed and relatively uniform in size. The hydrodynamic diameter of CuS NPs was determined to be 28 nm using DLS.

For further comparative tests involving cell or in vivo experiments, the stability of CuS NPs is critical. PEG is one of the most commonly used stabilizing ligands as it can provide nanoparticles with steric hindrance to maintain their dispersity in a biological environment and thus achieving extended circulation life, increased stability, and protection against detection and degradation by the immune system [26, 28, 29]. Therefore, PEG was chosen for the modification and stabilization of CuS NPs. The average hydrodynamic diameter of the resulted CuS-PEG NPs is about 57 nm (Fig. 1b), which is 29 nm larger than that of CuS NPs. The average apparent zeta potential of CuS-PEG NPs is − 13.2 mV, which is 14 mV higher than that of CuS NPs (Fig. 1c). Subsequently, the stability of CuS-PEG NPs in FBS was monitored. The nanoparticle size did not show significant change, which proved that CuS-PEG NPs were stable in FBS for 7 days (Fig. 1d). Photoacoustic (PA) effect of CuS-PEG NPs was also determined. As shown in Fig. 1e, the intensity of PA signal was proportional to the concentration of CuS-PEG NPs. The *R*^2^ value was calculated to be 0.993. Absorption spectrum measurement (from 400 to 1350 nm) (Fig. 1f) showed that the absorption values at 808 and 1275 nm were close which were 1.33 and 1.08, respectively. The maximum absorption peak of the CuS-PEG NPs prepared located at ~ 1000 nm. Therefore, the CuS-PEG NP was chosen to act as a nanoplatform for the comparative studies between 808 and 1275 nm laser. The concentration of the samples was adjusted to make up the absorption difference.

### Tissue Penetration Capabilities Ex Vivo

**Fig. 2:**
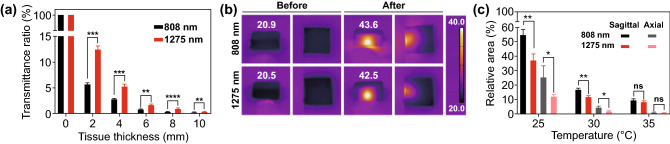
Transmittance and scattering effect of 808 and 1275 nm laser to porcine muscle. **a** Quantitative result of remained intensity ratio through porcine muscle of different thicknesses. **b** Thermal images of porcine muscle tissues after 808 or 1275 nm laser irradiation and **c** quantitative results of the area above certain temperatures. *p < 0.05, **p < 0.001, ***p < 0.001, ****p < 0.0001; Student’s *t* test. The error bars represent standard deviations (*n* = 3 per group)

Theoretically, NIR-II window is more desirable than the traditional NIR-I window owing to the much deeper tissue penetration [30]. Porcine muscles with different thicknesses were used to mimic deep-tissue environment in living subject. Comparison of laser transmittance through tissues was carried out. As shown in Fig. 2a, 1275 nm laser illumination exhibited much higher transmittance ratios than those of 808 nm laser under the same power density and tissue depth. For the testing group with 2-mm porcine muscle block, the transmittance ratio of 1275 nm laser was 2.2-fold of that of 808 nm laser. The transmittance superiority of 1275 nm over 808 nm laser is partly due to the less tissue scattering effect of 1275 nm light. Another reason is that compared with 808 nm, 1275 nm has reduced absorption by hemoglobin, melanin, and other human tissues [31].

To test the scattering effect of tissue to 808 and 1275 nm laser, we visualized it by monitoring the temperature distribution of porcine muscle after the laser irradiation. Porcine muscles (2.5 cm long, 2.5 cm wide, and 0.8 cm in thickness) were laid flat and exposed to 808 or 1275 nm laser. Thermal images were taken before and after irradiation by infrared camera. The power of both lasers was adjusted, so that the porcine muscle could be heated to similar temperature. As shown in Fig. 2b, the center temperature of the porcine muscle in 808 nm group increased from 21.1 to 42.7 °C, while in 1275 nm group, it increased from 21.3 to 42.4 °C. The temperature increments in two groups were at similar level. Then, the relative area of the region where temperature was above a certain value was plotted (Fig. 2c). It was found that the sample in 808 nm group had a more diffuse distribution of temperature increment, while in 1275 nm group, the same temperature area distributed in a focused region. The area where temperature was above 30 °C of the axial side of 1275 nm group was only 43% of that in 808 nm group. These differences are related to the different scattering effects of porcine muscle tissue to laser at different wavelengths. Porcine muscle had a stronger scattering effect to 808 nm laser than that to 1275 nm laser. This finding is in consistence with the previous results in the literature that the scattering coefficients of several human tissues decrease when laser wavelength increases from 600 to 1300 nm [14]. Significantly, the tissue scattering effect to 1275 nm laser was much lower than that to 808 nm laser, which allowed PTT with 1275 nm laser to cause localized temperature rise, thus improving the spatial control accuracy of PTT.

### Deep-Tissue Photothermal Heating Capabilities

**Fig. 3:**
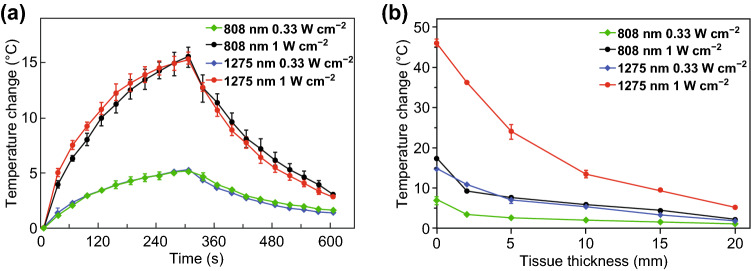
Temperature rising experiments in vitro. **a** Heating effects of 808 and 1275 nm laser to CuS-PEG NPs without tissue blocking. **b** Temperature rise of CuS-PEG NPs solution covered with different thicknesses of porcine muscle tissues under different laser excitation conditions. The error bars represent standard deviations (*n* = 3 per group)

The temperature changes of CuS-PEG NPs were recorded under the continuous laser irradiation of 0.33 or 1 W cm^−2^ of two lasers for 5 min. It was found that under the same laser power density, temperature rise of the 808 nm group was at the same level as that of the 1275 nm group (Fig. 3a). This indicated that without tissue blocking, when CuS-PEG NPs (0.25 mg mL^−1^ for 808 nm group and 0.30 mg mL^−1^ for 1275 nm group) were irradiated by 808 or 1275 nm laser with the same power density, the photothermal effects of these two CuS-PEG NPs by the two lasers were almost the same. A fair platform for the comparison between 808 and 1275 nm laser in deep-tissue photothermal capabilities had thus been established.

We further compared the deep-tissue photothermal heating capabilities of 808 and 1275 nm laser using porcine muscle tissues of different thicknesses (2, 5, 10, 15, and 20 mm) to mimic the deep-tissue environment. The MPE limit for skin for lasers of 808 nm laser is 0.33 W cm^−2^, while that of 1275 nm laser is 1 W cm^−2^ [32]. Therefore, these two power densities were chosen for the experiments. As shown in Fig. 3b, under the same laser power density, the temperature increments in CuS-PEG NPs solutions under 1275 nm irradiation were significantly higher than that under 808 nm groups. Moreover, if we compare the photothermal effect of 808 and 1275 nm laser under their MPE power density, the temperature increments in CuS-PEG NPs solution irradiated by 1275 nm laser at tissue depths of 2, 5, 10, 15, and 20 mm were 10.5-, 9.1-, 6.5-, 6.2-, and 4.8-fold of that by 808 nm laser. This further demonstrated the superiority of 1275 nm over 808 nm laser in photothermal capability for PTT, especially in treatment of deep-tissue tumors.

On the one hand, 1275 nm laser possesses deeper tissue penetration capability than 808 nm laser. The penetration capability of lasers to tissue depends upon the absorption and scattering effect of tissues to lasers of various wavelengths [33]. Biological tissues such as oxyhemoglobin, deoxyhemoglobin and fat absorb and scatter less photons of longer wavelength. The lower extinction coefficients of these contents contribute to the excellent penetration capability of lasers in the NIR-II region [4, 11, 34, 35]. On the other hand, the MPE limit of 1275 nm is higher than that of 808 nm. This is due to the lower photon energy at the longer wavelength [26]. It is worth pointing out that the power density used in most researches on PTT applying 808 nm laser is much higher than the MPE limit of 0.33 W cm^−2^ for 808 nm laser. This could raise safety concerns in their future clinical translation.

### Deep-Tissue Photothermal Capabilities In Vitro

**Fig. 4:**
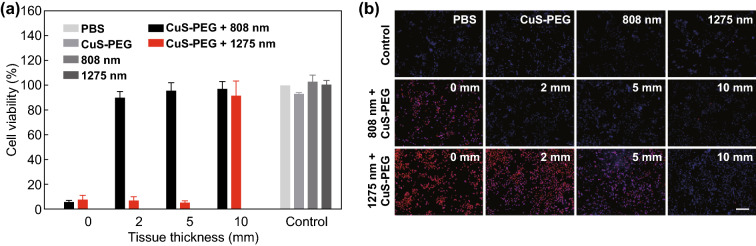
Comparison of photothermal capabilities in vitro. **a** Cell viability after photothermal treatments covered with porcine muscle tissues of varying thicknesses. **b** Fluorescence micrographs of cells after Hoechst PI staining. The scale bar is 200 μm. The error bars represent standard deviations (*n* = 3 per group)

Photothermal capabilities of 808 and 1275 nm lasers in tissue-mimicking phantoms were further compared in vitro. 4T1 cells were covered with a series of porcine muscle tissues and incubated with CuS-PEG NPs. Then, 808 or 1275 nm laser was applied for 5 min. Finally, cell viability was assessed through the 3-(4,5-dimethylthiazol2-yl)-2,5-diphenyltetrazolium bromide (MTT) assay. As shown in Fig. 4a, CuS-PEG NPs or laser irradiation alone exhibited little effect on cell viability. CuS-PEG NPs plus 1275 nm laser irradiation showed prominent photothermal killing of 4T1 cells with covering porcine muscle tissues of up to 5 mm thickness, while CuS-PEG NPs plus 808 nm laser irradiation only efficiently killed cells without tissue covering. These results supported the notion that 1275 nm laser was superior to 808 nm laser in photothermal capability in deep-tissue treatment applications.

Fluorescence microscopy was also performed to evaluate cell treatment in different groups. Blue fluorescence Hoechst 33,342 and red fluorescence PI were used to distinguish living cells, apoptotic cells, and necrotic cells, respectively (Fig. 4b). Weak blue fluorescence was observed in all control groups, including incubation with PBS or CuS-PEG NPs or irradiation by 808 or 1275 nm laser, which indicated that CuS-PEG NPs or laser irradiation nearly did no harm to 4T1 cells when they were applied alone. Both strong blue and red fluorescence appeared in cells of “CuS-PEG NPs + 808 nm” group or “CuS-PEG NPs + 1275 nm + 5 mm porcine muscle tissue” group, revealing that both apoptosis and necrosis occurred. Cells in the “CuS-PEG NPs + 1275 nm” group without tissue covering showed red fluorescence, indicating that most cells were necrotic. These results clearly demonstrated that 1275 nm was superior to 808 nm for PTT in both penetration and efficacy.

### Deep-Tissue Antitumor Capabilities In Vivo

**Fig. 5:**
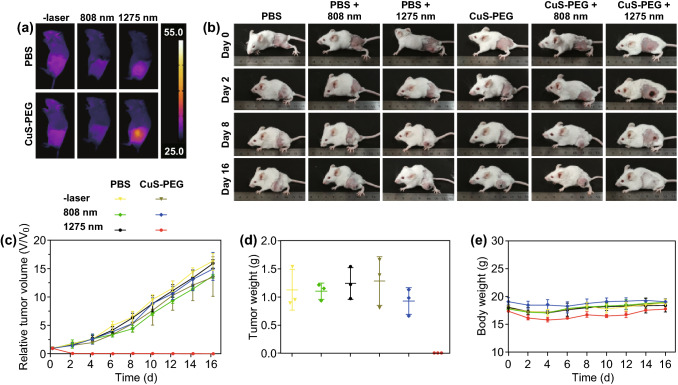
Comparison of antitumor capabilities in vivo. **a** Infrared thermal image of mice after different treatments. **b** Representative pictures of mice after different treatments. **c** Relative tumor size of mice after different treatments. **d** Weights of tumors in mice sacrificed after 16-day observation. **e** Body weights of mice after different treatments. The error bars represent standard deviations (*n* = 3 per group)

Comparison of the antitumor capabilities in deep-tissue environment between 808 and 1275 nm lasers in vivo was carried out using 4T1 tumor-bearing mice models. PBS or CuS-PEG NPs (50 μL) were intratumorally injected into tumors. Deep-tissue environment was mimicked by covering 5-mm-thick porcine muscle tissues on the top of the tumors. Different lasers of 1 W cm^−2^ were applied for 5 min. Infrared thermal images of mice captured after different treatments are shown in Fig. 5a. The temperatures of PBS-injected tumor irradiated by 808 or 1275 nm laser were 29.8 and 36.2 °C, respectively, which were both below the threshold temperature required to induce tumor destruction (43 °C). Meanwhile, the temperature of CuS-PEG NPs-injected tumor irradiated by 1275 nm laser reached 46.8 °C, which was 1.6-fold of that irradiated by 808 nm laser (29.8 °C). The pictures of mice treated with different conditions on day 0, 2, 8, and 16 are shown in Fig. 5b, clearly exhibiting the outcomes of different treatments. Also, tumor volumes (Fig. 5c) and body weights (Fig. 5e) of mice were continuously recorded every 2 days in the following 16 days. The mice were sacrificed and tumor weights were also recorded on day 16. As shown in Fig. 5c, d, tumors injected with CuS-PEG NPs only or irradiated by 1275 nm laser only showed quick growth similar to the PBS-injected ones, indicating that CuS-PEG NPs or 1275 nm laser irradiation alone was not able to inhibit tumor growth. Tumor with PBS injection expanded as large as 16.3 times to its volume at the beginning of the therapy. Tumors injected with CuS-PEG NPs or PBS irradiated by 808 nm laser also grew nearly as fast as those treated with PBS alone. In contrast, tumors injected with CuS-PEG NPs irradiated by 1275 nm laser were totally ablated after the treatment. Among all these treatments, only 1275 nm laser irradiation along with intratumoral injection of CuS-PEG NPs displayed high antitumor efficacy. Moreover, no significant weight loss of mice was observed for all groups (Fig. 5e), indicating the safety of all treatments during the period of time monitored. Thus, compared to 808 nm laser, 1275 nm laser provided more efficient antitumor capability in PTT for tumor tissues in depth.

This superiority should be attributed to the deeper tissue penetration of 1275 nm laser, which had been proved by comparative experiments in vitro. In addition, according to the skin-tolerance threshold set by the America National Standards Institute, 808 nm laser has a MPE limit of 0.33 W cm^−2^, while that of 1275 nm laser is 1.0 W cm^−2^. The MPE regulation also limits the application of 808 nm laser for deep-tissue PTT. Therefore, the intrinsic merits of NIR-II light over NIR-I light that 1275 nm laser possesses deeper tissue penetration and higher MPE limit, contributes to the superiority of 1275 nm laser than 808 nm laser in deep-tissue PTT.

### PTT with 1275 nm Laser

**Fig. 6:**
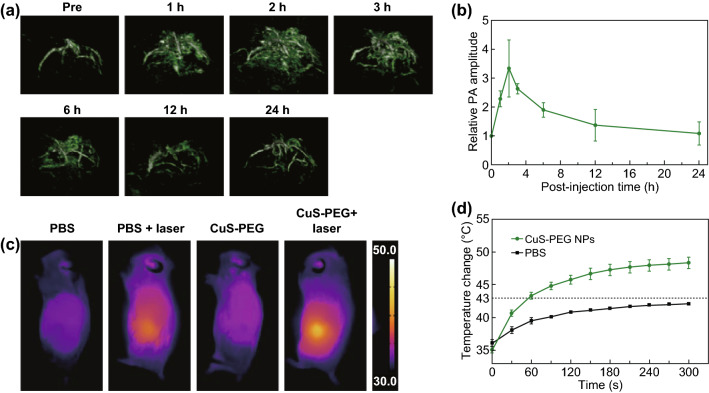
PAI of tumors and PTT with 1275 nm laser. **a** Three-dimensional reconstructed PA images of the tumor site before and in different time points after intravenous injections of CuS-PEG NPs (200 μL, 2 mg mL^−1^) and **b** quantitative results of relative PA signals. **c** Infrared thermal images of 4T1 tumor-bearing mice after different treatments. **d** Tumor temperature as a function of laser irradiation time after intravenous injection of CuS-PEG NPs (200 μL, 2 mg mL^−1^) or PBS (200 μL). The error bars represent standard deviations (*n* = 3 per group)

To further evaluate the efficacy of the 1275 nm laser for PTT, CuS-PEG NPs were intravenously injected to 4T1 tumor-bearing mice. PTT with 1275 nm laser was conducted. To identify the optimal therapeutic window, PA signals of CuS-PEG NPs accumulated in the tumors were monitored through photoacoustic imaging (PAI), an emerging biomedical imaging technology combining the high contrast of optical imaging and the high spatial resolution of ultrasonic imaging [36–38]. CuS-PEG NPs accumulated in the tumor due to the EPR effect. The abnormally tortuous and leaky tumor vasculature combined with poor lymphatic drainage caused heightened accumulation of long-circulating macromolecules [39–41]. Three-dimensional reconstructed PA images were obtained before and after 1, 2, 3, 6, 12, and 24 h of intravenous injection of CuS-PEG NPs. The average PA amplitudes at different times are shown in Fig. 6a. It was noticed that at 2-h post-injection, PA signals reached the maximum level.

PTT was carried out at 2-h post-injection. The surface temperature changes of tumor during irradiation are recorded and shown in Fig. 6d. The infrared thermal images of mice of the different group right after irradiation are captured and shown in Fig. 6c. In the “CuS-PEG NPs + laser” group, tumor temperature raised to 46.9 °C, which was above the effective PTT threshold temperature of 43 °C. In comparison, tumor in the “PBS + laser” group only raised to 42.3 °C. The temperature increment in this group would give credit to the abundant blood flow in 4T1 tumor compared to surrounding tissue.

### Outcome Evaluation of PTT with 1275 nm Laser

**Fig. 7:**
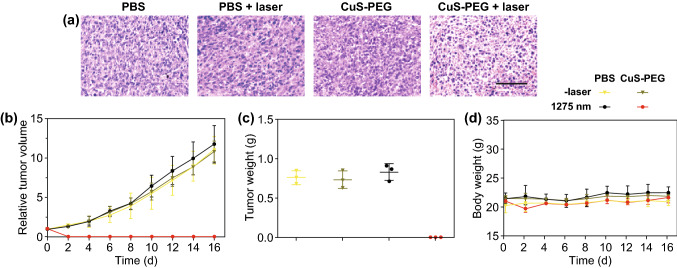
Histological analysis and records of tumor volume and mice weight. **a** H&E stained results of tumor sections that were collected from mice of different treatments. The scale bar is 100 μm. **b** Tumor growth data of different groups of mice after different treatments. **c** Weights of tumors in mice sacrificed after 16-day observation. **d** Body weight data of different groups of mice after treatments. The error bars represent standard deviations (*n* = 3 per group)

After photothermal treatment, mice were sacrificed. Hematoxylin and eosin (H&E) staining analysis was performed to evaluate the histopathologic damage of tumors (Fig. 7a), and ICP-OES analysis was conducted to detect the distribution of Cu in normal tissues at 2-h post-injection (Fig. S4). No evident histopathological damage was observed in tumors in “CuS-PEG NPs” or “PBS + laser” group, while obvious histopathological changes were observed in tumors of mice treated with intratumoral injection of CuS-PEG NPs with laser irradiation, including cell shrinking, chromatin condensation, and tissue extracellular matrix corruption. These results demonstrated that 1275 nm laser caused efficient destruction to tumors with intravenous injection of CuS-PEG NPs.

Tumor volumes (Fig. 7b) and body weights (Fig. 7d) of mice were continuously monitored after different treatments to quantitatively evaluate the PTT efficacy. After monitoring the tumor volumes and body weights, we sacrificed the mice and weighted the tumors (Fig. 7c). Among all groups, mice weight did not show significant loss, which indicated the safety of different treatments during the period of this study. Tumors of mice treated with CuS-PEG NPs or laser alone exhibited quick growth trends similarly to PBS treated ones, which expanded about 12-fold on day 16. Only the group treated with CuS-PEG NPs plus laser irradiation showed significant ablation of tumors.

It is worth noting that the power density used in this treatment was only 0.2 W cm^−2^, which was much lower than the 1 W cm^−2^ in most current researches. By reducing the power density used in PTT, the safety of the treatment is expected to be promoted. Besides, the temperature increment in this therapy was mild. The high temperature in PTT not only kills cancer cells but also does damage to the surrounding normal tissue by heat conduction, which decreases the therapeutic accuracy and brings about casualties [42]. Therefore, applying an appropriate laser dosage to achieve desirable PTT outcomes and reducing damage to normal tissue are of great importance to the clinical application of PTT. Lasers with a relatively low energy and improved penetration not only suffice for the requirement of PTT, but also reduce potential safety concerns.

## Conclusion

In conclusion, the capacity of 1275 nm laser in PTT has been thoroughly evaluated in this study. Moreover, the deep-tissue PTT efficacy of 1275 nm laser has also been compared with NIR-I laser which represented by 808 nm laser. Our results show that 1275 nm laser is more efficient than 808 nm laser to cause temperature rise in vitro and tumor destruction in vivo. PTT with 1275 nm laser after intravenous injection of CuS-PEG NPs exhibits excellent ability for ablation of tumor. NIR-IIa 1275 nm laser would be a promising candidate for clinical translations of PTT.

## Electronic supplementary material

Below is the link to the electronic supplementary material.

Supplementary material 1 (PDF 405 kb)

## References

[1] He S, Song J, Qu J, Cheng Z (2018). Crucial breakthrough of second near-infrared biological window fluorophores: design and synthesis toward multimodal imaging and theranostics. Chem. Soc. Rev..

[2] Abbas M, Zou Q, Li S, Yan X (2017). Self-assembled peptide- and protein-based nanomaterials for antitumor photodynamic and photothermal therapy. Adv. Mater..

[3] Hu JJ, Cheng YJ, Zhang XZ (2018). Recent advances in nanomaterials for enhanced photothermal therapy of tumors. Nanoscale.

[4] Ge X, Fu Q, Bai L, Chen B, Wang R (2019). Photoacoustic imaging and photothermal therapy in the second near-infrared window. New J. Chem..

[5] Li J, Pu K (2019). Development of organic semiconducting materials for deep-tissue optical imaging, phototherapy and photoactivation. Chem. Soc. Rev..

[6] Lin H, Gao S, Dai C, Chen Y, Shi J (2017). A two-dimensional biodegradable niobium carbide (MXene) for photothermal tumor eradication in NIR-I and NIR-II biowindows. J. Am. Chem. Soc..

[7] Tsai MF, Chang SH, Cheng FY, Shanmugam V, Cheng YS (2013). Au nanorod design as light-absorber in the first and second biological near-infrared windows for in vivo photothermal therapy. ACS Nano.

[8] Cao Z, Feng L, Zhang G, Wang J, Shen S (2018). Semiconducting polymer-based nanoparticles with strong absorbance in NIR-II window for in vivo photothermal therapy and photoacoustic imaging. Biomaterials.

[9] Sun T, Dou JH, Liu S, Wang X, Zheng X (2018). Second near-infrared conjugated polymer nanoparticles for photoacoustic imaging and photothermal therapy. ACS Appl. Mater. Interfaces.

[10] Sun T, Han J, Liu S, Wang X, Wang ZY (2019). Tailor-made semiconducting polymers for second near-infrared photothermal therapy of orthotopic liver cancer. ACS Nano.

[11] Smith AM, Mancini MC, Nie S (2009). Bioimaging: second window for in vivo imaging. Nat. Nanotechnol..

[12] Cao Y, Dou J-H, Zhao N-J, Zhang S, Zheng Y-Q (2016). Highly efficient NIR-II photothermal conversion based on an organic conjugated polymer. Chem. Mater..

[13] Park J-E, Kim M, Hwang J-H, Nam J-M (2017). Golden opportunities: plasmonic gold nanostructures for biomedical applications based on the second near-infrared window. Small Methods.

[14] Bashkatov AN, Genina EA, Kochubey VI, Tuchin VV (2005). Optical properties of human skin, subcutaneous and mucous tissues in the wavelength range from 400 to 2000 nm. J. Phys. D-Appl. Phys..

[15] Jiang Y, Li J, Zhen X, Xie C, Pu K (2018). Dual-peak absorbing semiconducting copolymer nanoparticles for first and second near-infrared window photothermal therapy: a comparative study. Adv. Mater..

[16] Guo B, Sheng Z, Hu D, Liu C, Zheng H (2018). Through scalp and skull NIR-II photothermal therapy of deep orthotopic brain tumors with precise photoacoustic imaging guidance. Adv. Mater..

[17] Zhou J, Jiang Y, Hou S, Upputuri PK, Wu D (2018). Compact plasmonic blackbody for cancer theranosis in the near-infrared II window. ACS Nano.

[18] Hong G, Diao S, Chang J, Antaris AL, Chen C (2014). Through-skull fluorescence imaging of the brain in a new near-infrared window. Nat. Photonics.

[19] Diao S, Blackburn JL, Hong G, Antaris AL, Chang J (2015). Fluorescence imaging in vivo at wavelengths beyond 1500 nm. Angew. Chem. Int. Ed..

[20] Zhang XD, Wang H, Antaris AL, Li L, Diao S (2016). Traumatic brain injury imaging in the second near-infrared window with a molecular fluorophore. Adv. Mater..

[21] Wan H, Yue J, Zhu S, Uno T, Zhang X (2018). A bright organic NIR-II nanofluorophore for three-dimensional imaging into biological tissues. Nat. Commun..

[22] White PF, Zafereo J, Elvir-Lazo OL, Hernandez H (2018). Treatment of drug-resistant fibromyalgia symptoms using high-intensity laser therapy: a case-based review. Rheumatol. Int..

[23] White PF, Cao X, Elvir Lazo L, Hernandez H (2017). Effect of high-intensity laser treatments on chronic pain related to osteoarthritis in former professional athletes: a case series. J. Mol. Biomark. Diagn..

[24] Zhou M, Zhang R, Huang M, Lu W, Song S (2010). A chelator-free multifunctional [^64^Cu]CuS nanoparticle platform for simultaneous micro-PET/CT imaging and photothermal ablation therapy. J. Am. Chem. Soc..

[25] Ku G, Zhou M, Song S, Huang Q, Hazle J (2012). Copper sulfide nanoparticles as a new class of photoacoustic contrast agent for deep tissue imaging at 1064 nm. ACS Nano.

[26] Liu Y, Bhattarai P, Dai Z, Chen X (2019). Photothermal therapy and photoacoustic imaging via nanotheranostics in fighting cancer. Chem. Soc. Rev..

[27] Song S, Xiong C, Zhou M, Lu W, Huang Q (2011). Small-animal PET of tumor damage induced by photothermal ablation with 64Cu-bis-DOTA-hypericin. J. Nucl. Med..

[28] Harris JM, Chess RB (2003). Effect of pegylation on pharmaceuticals. Nat. Rev. Drug Discov..

[29] Antaris AL, Chen H, Cheng K, Sun Y, Hong G (2016). A small-molecule dye for NIR-II imaging. Nat. Mater..

[30] Feng W, Han X, Wang R, Gao X, Hu P (2019). Nanocatalysts-augmented and photothermal-enhanced tumor-specific sequential nanocatalytic therapy in both NIR-I and NIR-II biowindows. Adv. Mater..

[31] Ash C, Town G, Clement M (2010). Confirmation of spectral jitter: a measured shift in the spectral distribution of intense pulsed light systems using a time-resolved spectrometer during exposure and increased fluence. J. Med. Eng. Technol..

[32] Smith M, Fork RL, Cole S (2001). Safe delivery of optical power from space. Opt. Express.

[33] Welsher K, Sherlock SP, Dai H (2011). Deep-tissue anatomical imaging of mice using carbon nanotube fluorophores in the second near-infrared window. Proc. Natl. Acad. Sci. U. S. A..

[34] Sordillo LA, Pu Y, Pratavieira S, Budansky Y, Alfano RR (2014). Deep optical imaging of tissue using the second and third near-infrared spectral windows. J. Biomed. Opt..

[35] Zhang Z, Suo H, Zhao X, Sun D, Fan L (2018). NIR-to-NIR deep penetrating nanoplatforms Y_2_O_3_:Nd(3^+^)/Yb(3^+^)@SiO_2_@Cu_2_S toward highly efficient photothermal ablation. ACS Appl. Mater. Interfaces.

[36] Wang LV, Hu S (2012). Photoacoustic tomography: in vivo imaging from organelles to organs. Science.

[37] Wu B, Lu ST, Yu H, Liao RF, Li H (2018). Gadolinium-chelate functionalized bismuth nanotheranostic agent for in vivo MRI/CT/PAI imaging-guided photothermal cancer therapy. Biomaterials.

[38] Zhang Y, Zhao N, Qin Y, Wu F, Xu Z (2018). Affibody-functionalized Ag_2_S quantum dots for photoacoustic imaging of epidermal growth factor receptor overexpressed tumors. Nanoscale.

[39] Jiang Y, Upputuri PK, Xie C, Zeng Z, Sharma A (2019). Metabolizable semiconducting polymer nanoparticles for second near-infrared photoacoustic imaging. Adv. Mater..

[40] Hong G, Antaris AL, Dai H (2017). Near-infrared fluorophores for biomedical imaging. Nat. Biomed. Eng..

[41] Blanco E, Shen H, Ferrari M (2015). Principles of nanoparticle design for overcoming biological barriers to drug delivery. Nat. Biotechnol..

[42] Zhu X, Feng W, Chang J, Tan YW, Li J (2016). Temperature-feedback upconversion nanocomposite for accurate photothermal therapy at facile temperature. Nat. Commun..

