# Supplementary material for: Deep-Tissue Photothermal Therapy Using Laser Illumination at NIR-IIa Window
Source: Nanomicro Lett. 2020 Jan 24;12:38. doi: 10.1007/s40820-020-0378-6 (PMC7770864; doi:10.1007/s40820-020-0378-6)
Supplement: Supplementary file 1 — Supplementary material 1 (PDF 405 kb) [file 40820_2020_378_MOESM1_ESM.pdf]

Supporting Informaiton for

Deep-tissue Photothermal Therapy Using Laser Illumination at NIR-IIa Window

Xunzhi Wu[1], Yongkuan Suo[1], Hui Shi[1], Ruiqi Liu[1], Fengxia Wu[1], Tingzhong Wang[2], Lina Ma[3], Hongguang Liu[1,*], Zhen Cheng[4,*]

[1]Institute of Molecular Medicine, College of Life and Health Sciences, Northeastern University, Shenyang 110000, People's Republic of China

[2]Department of Neurosurgery, the Fourth Affiliated Hospital of China Medical University, Shenyang 110000, People's Republic of China

[3]Changchun Institute of Applied Chemistry, Chinese Academy of Science, Changchun 130000, People's Republic of China

[4]Molecular Imaging Program at Stanford, Stanford University, Palo Alto, CA 94301, USA

*Correspongding authors. E-mail: simonliu@mail.neu.edu.cn (Hongguang Liu); zcheng@stanford.edu (Zhen Cheng)

Supplementary Data

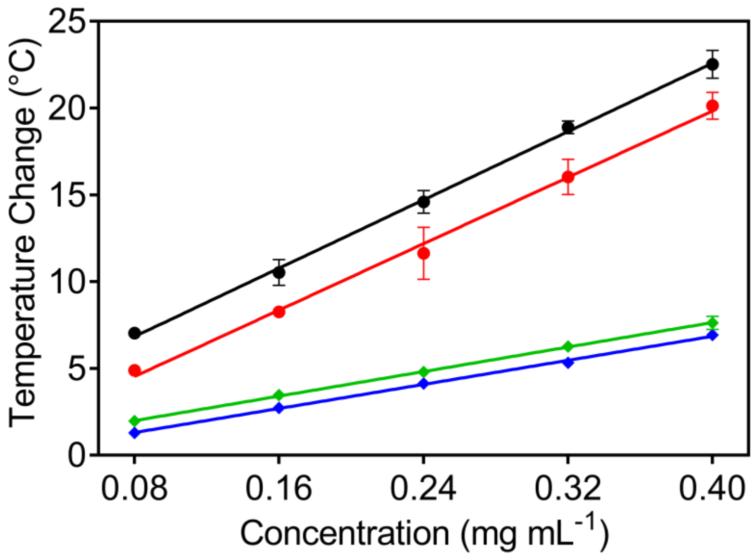

Fig. S1 Relationship between temperature rise and concentration of CuS-PEG NPs under 808 and 1275 nm laser

The relationships between temperature rise and concentration of CuS-PEG NPs under 808 and 1275 nm laser were determined. This is because the fairness of the comparison work is based on the condition that, without tissue blocking, the temperature rises of photothermal transduction agents excited by 808 or 1275 nm laser are similar. Though the absorption values at 808 and 1275 nm were close, small difference remains and would affect the fairness of the comparative experiment to a certain extent. To further improve the fairness of the comparative study, we first determined the relationship between the temperature increment and concentrations of CuS-PEG NPs solution under 808 and 1275 nm lasers. Deionized water and CuS-PEG NPs solution with gradient concentration (0.08, 0.16, 0.24, 0.32, and 0.40 mg mL^{-1}) were added in 96-well plates and then irradiated by 808 nm or 1275 nm laser with power density of 0.33 or 1 W cm^{-2} for 5 min. The temperature of each well was recorded by using an infrared thermal imaging instrument and the actual temperature rises of CuS-PEG NPs in each well were calculated by the temperature rise values of the solution minus those of the solvent, deionized water.

As shown in Fig. S1, within the concentration range of 0.08 to 0.40 mg mL^{-1}, the temperature increment of CuS-PEG NPs under 808 or 1275 nm laser had a linear relationship with its concentration. By analysis, it was found that, to achieve the same degree of temperature increment under the same laser power density, 0.30 mg mL^{-1} CuS-PEG NPs under 1275 nm irradiation was equivalent to 0.25 mg mL^{-1} NPs under 808 nm excitation. An increase of 20% in concentration for 1275 nm group was thus needed to make up its absorbance disadvantage compared to 808 nm group.

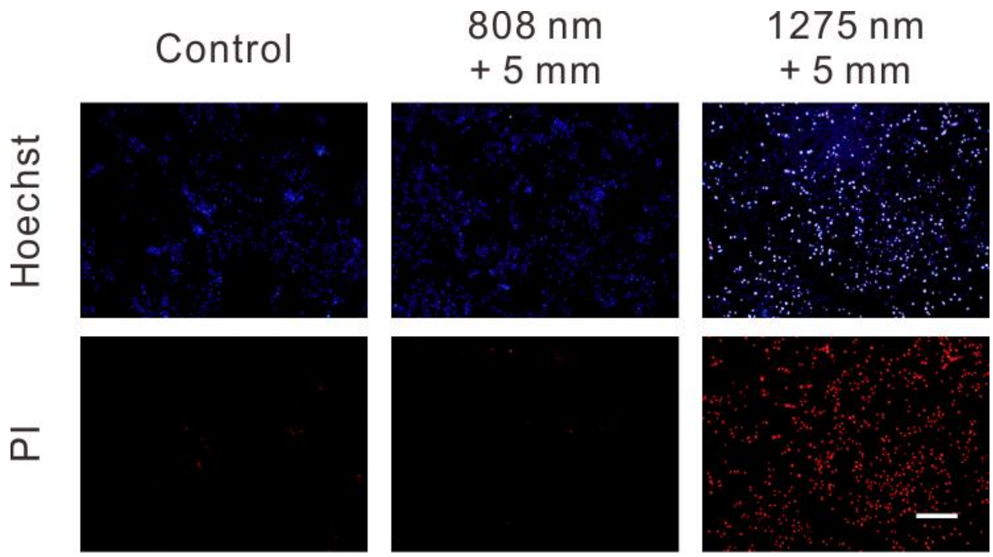

Fig. S2 Fluorescence micrographs of cells after Hoechst-PI staining

As shown in Fig. S2, weak blue fluorescence was observed in control group and “808 nm + 5 mm porcine muscle tissue” group, indicating the live state of cells. Strong blue and red fluorescence appeared in cells of “CuS-PEG NPs + 1275 nm + 5 mm porcine muscle tissue” group, revealing that both apoptosis and necrosis occurred. The scale bar is 200 μm.

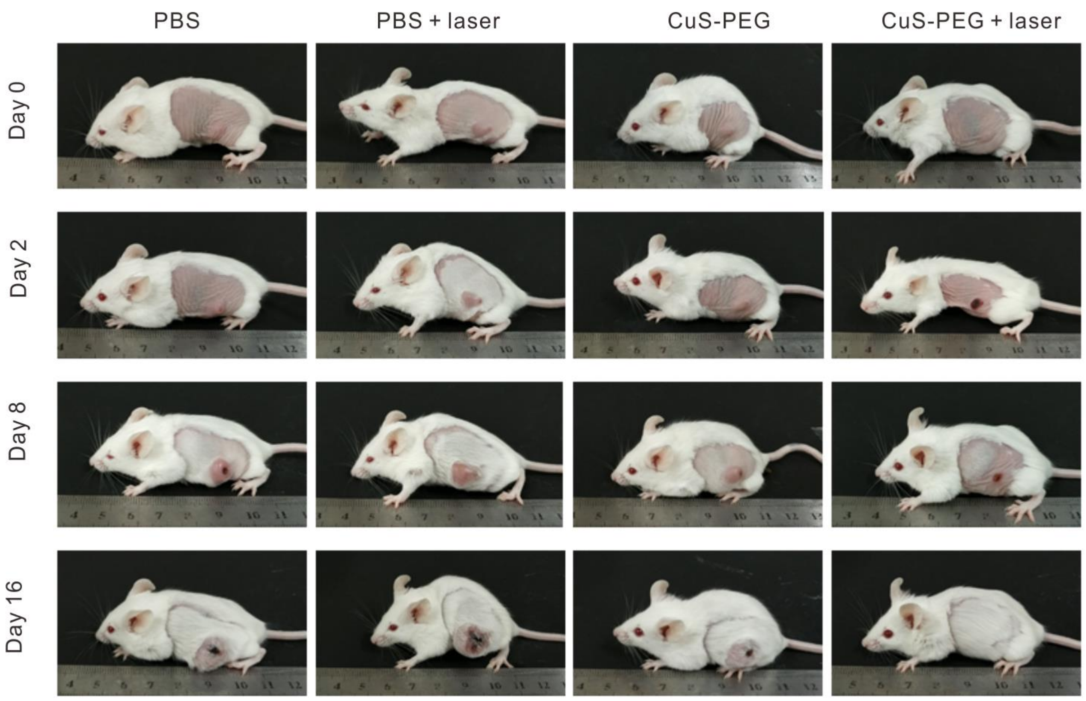

Fig. S3 Representative photographs of 4T1-tumor bearing mice after different treatments

As shown in Fig.S3, tumors of mice treated with CuS-PEG NPs only or irradiated by 1275 nm laser only both showed quick growth which is similar to the PBS-treated ones, indicating that CuS-PEG NPs or 1275 nm laser irradiation alone was not able to inhibit tumor growth. By contrast, tumors of mice with intravenous injection of CuS-PEG NPs and irradiation of 1275 nm laser were totally ablated.

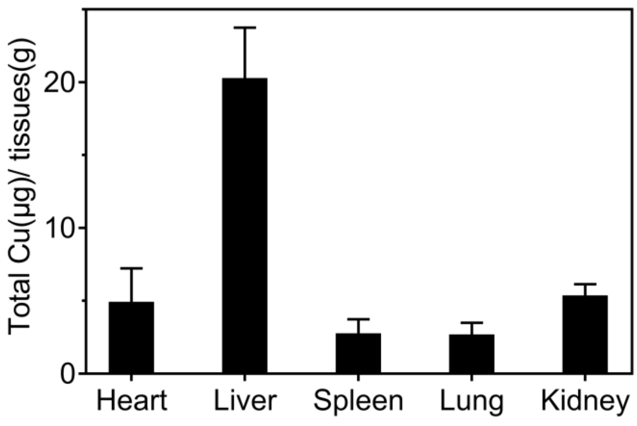

Fig. S4 Biodistribution of Cu in major organs including the heart, liver, spleen, lung and kidney determined by ICP-OES at 2 h post injection

The distribution of CuS-PEG NPs in normal tissue was evaluated by ICP-OES analysis at 2 h post injection. As shown in Fig. S4, significant accumulation was found in the liver, indicating that liver is the major organ for the metabolism and clearance of CuS-PEG NPs.
